# Health Behaviors of Korean Gastric Cancer Survivors with Hypertension: A Propensity Analysis of KNHANES III-V (2005–2012)

**DOI:** 10.1371/journal.pone.0126927

**Published:** 2015-05-22

**Authors:** So-Ra Jo, Ju-Youn Joh, Ju-Ri Jeong, Sun Kim, Yeon-Pyo Kim

**Affiliations:** 1 Department of Family Medicine, Hwasun Chonnam National University Hospital, Hwasun-eup, Hwasun-gun Chonnam, Korea; 2 Clinic of Life After Cancer Treatment (LACT Clinic), Hwasun Chonnam National University Hospital, Hwasun-eup, Hwasun-gun Chonnam, Korea; Beijing University of Chemical Technology, CHINA

## Abstract

**Objective:**

This study provides a comparison of health behaviors between gastric cancer survivors with hypertension and non-cancer subjects in Korea.

**Methods:**

Data from the Korean National Health and Nutrition Examination Survey (KNHANES) for the period of 2005-2012 were used in this study. A propensity score matching method was used to compare health behaviors. Before the matching of propensity scores, the number of participants was 11034 (102 gastric cancer survivors and 10932 non-cancer participants). A 1:5 propensity score matching procedure yielded a total of 480 participants (80 gastric cancer survivors and 400 non-cancer participants) for the final analysis. Drinking, smoking, physical activity, antihypertensive medication adherence, self-reported diet control, and sodium intake accordance in the two groups were compared. A complex samples logistic regression analysis was conducted to assess any differences between the two groups.

**Results:**

The group of hypertensive gastric cancer survivors had lower alcohol consumption (OR = 0.30; 95% CI: 0.14-0.66; p-value = 0.003). They were more likely to be on dietary control than the control group (OR = 3.12; 95% CI: 1.60-6.10; p-value = 0.001). However, there was no significant (p > 0.05) difference in sodium intake accordance or other health behaviors (including medication adherence, smoking, and physical activity) between the two groups.

**Conclusions:**

Our results revealed that gastric cancer survivors with hypertension were more likely to be on dietary control with lower alcohol consumption than the control group. However, there was no significant difference in sodium intake accordance or other health behaviors between the two groups. Therefore, primary care physicians should inform cancer survivors about the appropriate health behaviors to reduce their risk of cardiovascular disease and improve their overall survival rate, even though they say they have been doing health behaviors.

## Introduction

Because of advances in early-diagnosis and treatment techniques, the number of people living with cancer has been increasing [1]. According to Korea data for the period of 2005 to 2009, 62% of all cancer patients survived more than five years. Some types of cancer, including thyroid cancer (99.7%), breast cancer (90.6%), prostate cancer (87.6%), colon cancer (71.3%), and gastric cancer (65.3%), showed higher survival rates [2]. Between 1999 and 2010, there were 960,654 cancer survivors in Korea. Nearly a million people are expected to be cancer survivors in 2014. Cancer survivor means all people being alive after diagnosis with cancer [2], but there is a controversy about that. Cancer survivors have been recognized as a growing group. Their health problems and quality of life have become a topic of considerable interest among physicians.

According to the data from the Korean National Cancer Center (2012), 35.8% of cancer patients had diseases other than cancer. Almost half of cancer patients died from problems other than cancer [3], such as cerebrovascular disease (18.5%), diabetes (7.8%), cardiovascular disease (6.8%), and suicide (6.2%). There is no significant difference in causes of death between cancer survivors and the general population [3]. Hypertension is a well-known risk factor of cardiovascular disease. It is the most common chronic disease among cancer survivors regardless of the type of cancer [4]. Chronic diseases including hypertension and cancer share life-style risk factors such as smoking, dietary habit, lack of physical activity, and alcohol abuse [4]. This suggests the importance of cancer management as well as non-cancer-related health care for reducing the morbidity burden and improving their overall survival rate.

Previous studies have increasingly emphasized the importance of managing cancer survivors' chronic disease. Some have examined health behaviors of cancer survivors. However, such studies have produced mixed results. For example, some studies have suggested that cancer survivors may be more likely to change their health behaviors than the general population [5,6], whereas others have provided opposite findings [7–9].

Incidence rates of gastric cancer per 100,000 in Korea, Japan, and China are 65.9, 65.8, 34.1 in men and 25.9, 25.2, 17.2 in women [10]. This suggests the importance of gastric cancer in Asia. However, no study has considered health behaviors of gastric cancer survivors with a specific comorbidity. In this regard, the present study investigated their health behaviors, including their tendency to manage their chronic disease.

This study serves as a starting point for examining health behaviors of community dwelling gastric cancer survivors with specific comorbid conditions with the aim to provide primary care physicians more effective cancer survivorship care.

## Materials and Methods

### Study population

Data were obtained from KNHANES III (2005), IV (2007–2009), and V (2010–2012). KNHANES was a nationally representative cross-sectional survey conducted by the Korean Ministry of Health and Welfare. This survey employed a stratified multistage clustered probability sampling method to reflect the non-institutionalized Korean population. Participants completed questionnaire consisting of a health interview survey, a health behavior survey, a nutrition survey, and a health examination survey [11].

Individuals with other types of cancer and those under the age of 19 were excluded from the analysis. Only those individuals with history of hypertension were selected. Hypertension was defined as a repeatedly elevated blood pressure exceeding 140 over 90 mmHg (a systolic pressure above 140 or a diastolic pressure above 90). Participants were classified as cancer survivors if they reported that they had been diagnosed by a physician with gastric cancer. Their current cancer status could not be assessed because no data were collected in the KNHANES on cancer symptoms or cancer treatments. Hypertension group consisted of participants who were diagnosed with hypertension by their doctor before. The number of participants was 11,034 before propensity score matching, including 102 gastric cancer survivors and 10,932 non-cancer participants. After a 1:5 propensity score matching procedure, there were 480 participants, including 80 gastric cancer survivors and 400 non-cancer controls. The other twenty-two gastric cancer survivors were excluded due to missing data. The study protocol did not require any institutional review board approval because KNHANES data were publicly available.

### Measures

#### Sociodemographic characteristics and chronic conditions

Sociodemographic characteristics included age, gender, education level (≤ elementary school, middle school, high school, and ≥ college), marital status (single, married, and widowed/divorced/separated), household income (low, middle-low, middle-high, and high), and private insurance. Health-related characteristics included participant's height, body weight, total energy intake, and comorbidities (dyslipidemia, diabetes, ischemic heart disease, stroke, asthma, chronic obstructive pulmonary disease (COPD), arthritis, thyroid disease, and depression). These comorbities were considered present for those who had ever been diagnosed by a physician.

#### Health behaviors

This study assessed health behaviors for hypertensive patients (physical activity and diet) and medication adherence based on the 2013 American College of Cardiology/American Heart Association (ACC/AHA) Guideline on Lifestyle Management to Reduce Cardiovascular Risk [12]. In addition, health behaviors to reduce the mortality of cancer survivors were compared between the two groups, including smoking, alcohol consumption, physical activity, and diet.

The following health behaviors were assessed: alcohol consumption (yes: at least once a month in the past year; or no: fewer than once a month in the past year), smoking (current smokers or non-current smokers: those who never smoked and former smokers who quit smoking), self-reported diet control (yes: those who answered "yes" to a question "Do you control your diet due to special reasons?", or no), and physical activity (yes or no). Physical activity was measured through frequency (sessions per week) and duration (in minutes or hours) of each session. Subjects were considered physically active if they participated in moderate-to-vigorous aerobic physical activity three to four sessions per week and the activity lasted an average of 40 minutes per session. Taking antihypertensive medication was measured by frequency (per month). Good antihypertensive medication adherence was defined as daily medication. Levels below this were regarded as not meeting sufficient medication adherence. Dietary sodium intake was obtained based on their 24-hour recall in the KNHANES nutrition survey. According to the 2013 AHA/ACC guidelines, hypertensive patients are recommended to reduce dietary sodium intake to no more than 2,400 mg (6 g sodium chloride). Sodium intakes below 2,400 mg of participants were regarded as meeting the recommendation.

### Assembly of study cohort: propensity score matching

**Fig 1:**
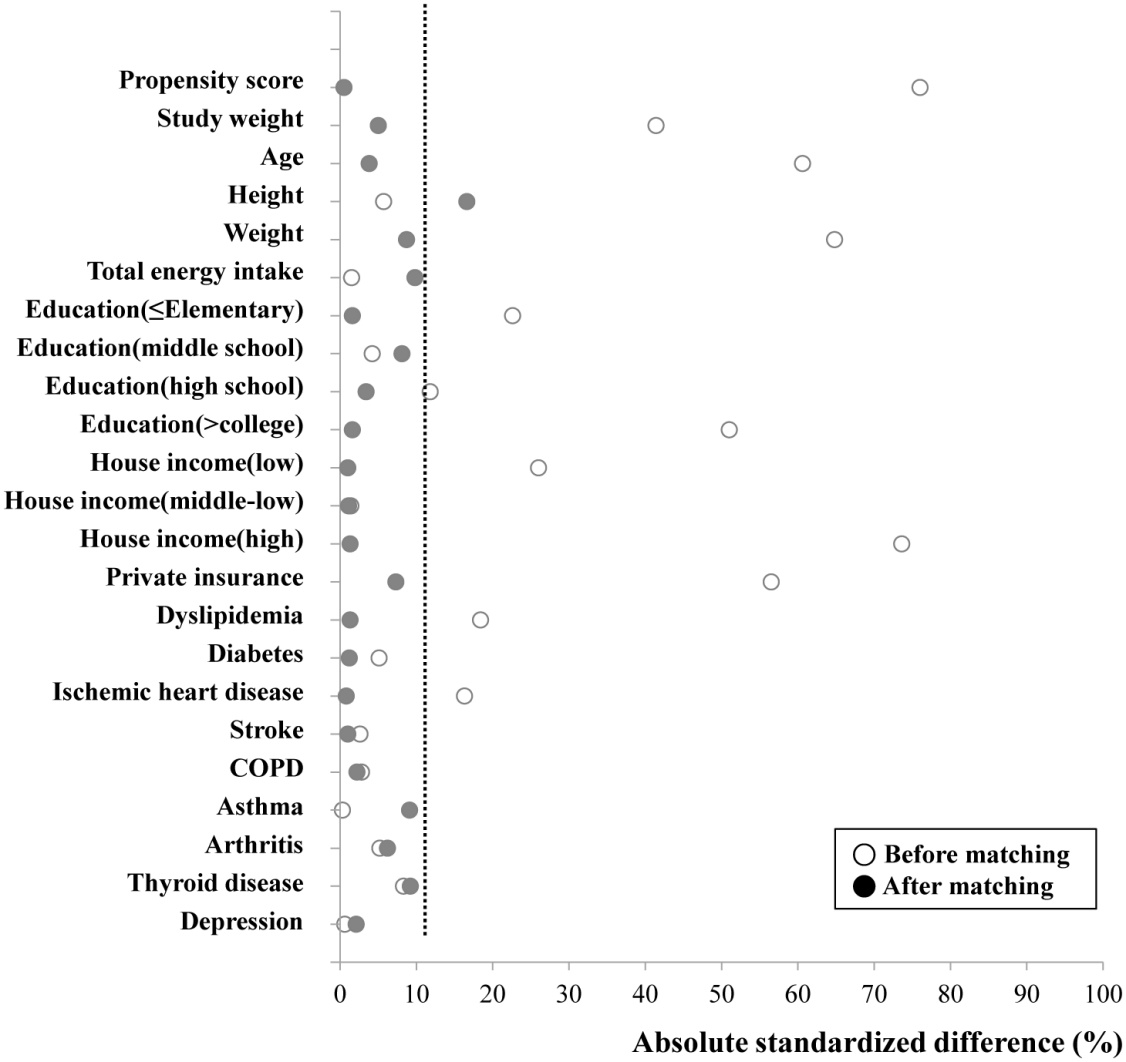
Absolute standardized differences. Absolute standardized differences comparing baseline characteristics of gastric cancer survivors with hypertension and non-cancer participants with hypertension before matching and after 1:5 propensity score matching. Y axis was the baseline characteristics. X axis of the scatterplot represented whether the status was before-matching or after matching.

Considering that the balance between the two groups achieved by randomization may be lost, we used propensity score approach to assemble a cohort and control for any imbalance in confounding factors in gastric cancer survivors with hypertension and non-cancer individuals with hypertension. The propensity score for an individual defined as the conditional probability of being the case group given the individual’s covariates is reported to be able to balance covariates in the two groups, and thus reduce bias [13]. Propensity scores were calculated for each of the 80 cancer survivors and 7173 non-cancer participants who were hypertensive adults without missing value of variables through multivariable logistic regression analysis based on all baseline characteristics listed in Table 1 as covariates. After estimating the propensity score, our sample sizes of the cancer and non-cancer participants varied greatly. Therefore one to five matching was performed [14]. The nearest available matching based on estimated propensity scores was performed with an application program in SPSS in order to select for the most similar propensity score in a 1:5 ratio [13–15]. Absolute standardized differences were estimated to evaluate the pre-match imbalance and post-match balance and presented as a Love plot (Fig 1) [16]. An absolute standardized difference of 0% indicated no residual bias. Differences less than 10% were considered inconsequential [17]. Because substantial differences in height between matched participants remained after matching, we conducted additional regression adjustment to reduce such difference [18].

**Table 1:** Demographic and health-related characteristics of hypertensive participants by their cancer status based on KNHANES^1^ III (2005), IV (2007–2009), and V (2010–2012).

Characteristics	Cancer^3^	Noncancer^3^	P-value^4^
**Sample size (n)**	102	10932	
**Men**	62(62.8)	4591(46.7)	0.008
**Age (year)**	69.3±1.13	61.3±1.15	<0.001
**Height (cm)**	158.5±1.27	159.7±1.28	0.359
**Body weight (kg)**	56.1±1.47	64.4±1.48	<0.001
**Total energy intake (kcal/day)**	1674.78±92.57	1795.48±94.48	0.202
**Study weight**	698.92±69.34	250.51±71.40	<0.001
**Education**			
**≤Elementary school**	64(63.6)	5563(48.3)	0.017
**Middle school**	16(16.8)	1563(15.1)	
**High school**	17(16.6)	2266(23.6)	
**≥College**	4(3.0))	1187(13.0)	
**Marital status**			
**Single**	0(0)	183(2.7)	0.404
**Married**	72(72.7)	7619(72.1)	
**Widowed/divorced/separated**	30(27.3)	2888(25.3)	
**Household income**			
**Low**	50(47.6)	3832(32.3)	0.004
**Middle-low**	22(29.9)	2687(25.9)	
**Middle-high**	21(17.1)	2088(20.9)	
**High**	7(5.4)	1906(20.9)	
**Private insurance**			
**Yes**	25(24.5)	4175(51.1)	<0.001
**No**	70(75.5)	4916(48.9)	
**Comorbidity**			
**Dyslipidemia**	13(12.4)	2032(21.7)	0.098
**Diabetes**	26(22.0)	2224(20.1)	0.682
**Ischemic heart disease**	14(9.7)	725(6.0)	0.125
**Stroke**	6(4.9)	763(6.2	0.601
**Asthma**	6(4.2)	534(4.4)	0.921
**COPD** ^**2**^	2(0.9)	146(1.0)	0.926
**Arthritis**	28(27.6)	3154(25.0)	0.623
**Thyroid disease**	5(2.4)	413(3.7)	0.376
**Depression**	5(2.3)	612(5.5)	0.076

^1^KNHANES: The Korea National Health and Nutrition Examination Survey.

^2^COPD: Chronic obstructive pulmonary disease.

^3^Results were expressed as the mean ± standard error (SE) or unweighted numbers (weighted %).

Percentages were weighted using NHANES sampling weights.

^4^P-values were obtained from the complex samples crosstabs and the complex samples general linear model.

### Statistical analysis

All statistical analyses were conducted using SPSS complex sample procedure because KNHANES data set was selected through a representative, stratified, and clustered sampling method, not a random sampling method. Sociodemographic characteristics of participants were evaluated as un-weighted numbers and weighted percentages for categorical data. Means and standard errors were used to describe continuous data. Descriptive statistics and Pearson chi-square tests were used to examine sociodemographic characteristics and differences between gastric cancer survivors and noncancer controls. After propensity score matching, a logistic regression analysis was conducted to investigate any differences in health behaviors between gastric cancer survivors and propensity-matched controls using two different approaches: unadjusted and multivariable-adjusted after entering age, gender, height, and significant covariate (p < 0.1) in univariate analysis. For more accurate comparison, univariate analysis was conducted to select significant covariates for results. Only covariates significant (p < 0.1) were included in adjustment. Statistical significance was assumed at p < 0.05. SPSS 22.0.0.1 (IBM Co, Armonk, NY, USA) was used in all statistical analyses. For propensity matching, we used the propensity matching add-on for SPSS (Propensity score matching for SPSS, version 3.0.2).

## Results

### Characteristics of study groups

Demographic and health-related characteristics of the group of gastric cancer patients with hypertension and the control group are summarized in Table 1 (before matching) and Table 2 (after 1:5 propensity score matching). Our propensity score matching reduced standardized differences for most observed covariates below 10% in absolute value, except height (Fig 1). It demonstrated substantial improvement in covariate balance across the groups. Before matching, there were significant differences between the two groups in terms of age, gender, body weight, household income, study weight, education level, and private insurance (Table 1). But after matching, they were not significant different between the two groups (Table 2).

**Table 2:** Demographic and health-related characteristics after propensity matching.

Characteristics	Cancer^2^	Noncancer^2^	P-value^3^
**Sample size (n)**	80	400	
**Men**	47(61.6)	235(62.0)	0.949
**Age (year)**	69.2±1.26	69.0±1.46	0.897
**Height (cm)**	158.1±1.32	160.4±1.44	0.103
**Body weight (kg)**	55.9±1.56	58.5±1.68	0.127
**Total energy intake (kcal/day)**	1704,18±87.81	1860.63±128.40	0.223
**Study weight**	728.52±75.38	724.90±82.39	0.965
**Education**			
**≤Elementaryschool**	52(65.6)	246(61.5)	0.924
**Middle school**	13(14.3)	64(14.9)	
**High school**	13(17.5)	81(20.8)	
**≥College**	2(1.8)	9(0.8)	
**Marital status**			
**Single**	0(0)	5(1.4)	0.643
**Married**	57(72.2)	289(73.0)	
**Widowed/divorced/separated**	23(27.8)	106(25.6)	
**Household income**			
**Low**	40(46.2)	190(43.9)	0.440
**Middle-low**	20(33.3)	102(26.0)	
**Middle-high**	16(4.5)	93(25.7)	
**High**	3(3.9)	15(4.4)	
**Private insurance**			
**Yes**	16(22.2)	90(28.1)	0.418
**No**	59(77.8)	310(71.9)	
**Comorbidity**			
**Dyslipidemia**	13(14.3)	60(12.4)	0.693
**Diabetes**	19(21.7)	85(23.6)	0.735
**Ischemic heart disease**	10(8.4)	46(12.0)	0.350
**Stroke**	5(5.3)	37(8.6)	0.355
**Asthma**	4(3.0)	15(2.8)	0.914
**COPD** ^**1**^	1(0.3)	3(0.2)	0.670
**Arthritis**	21(28.2)	78(17.2)	0.056
**Thyroid disease**	5(2.8)	28(5.3)	0.155
**Depression**	5(2.6)	12(2.9)	0.827

^1^COPD: Chronic obstructive pulmonary disease.

^2^Results were expressed as the mean±standard error (SE) or unweighted numbers (weighted %).

Percentages were weighted using the NHANES sampling weights.

^3^P-values were obtained from the complex samples crosstabs and the complex samples general linear model.

### Health behaviors

**Fig 2:**
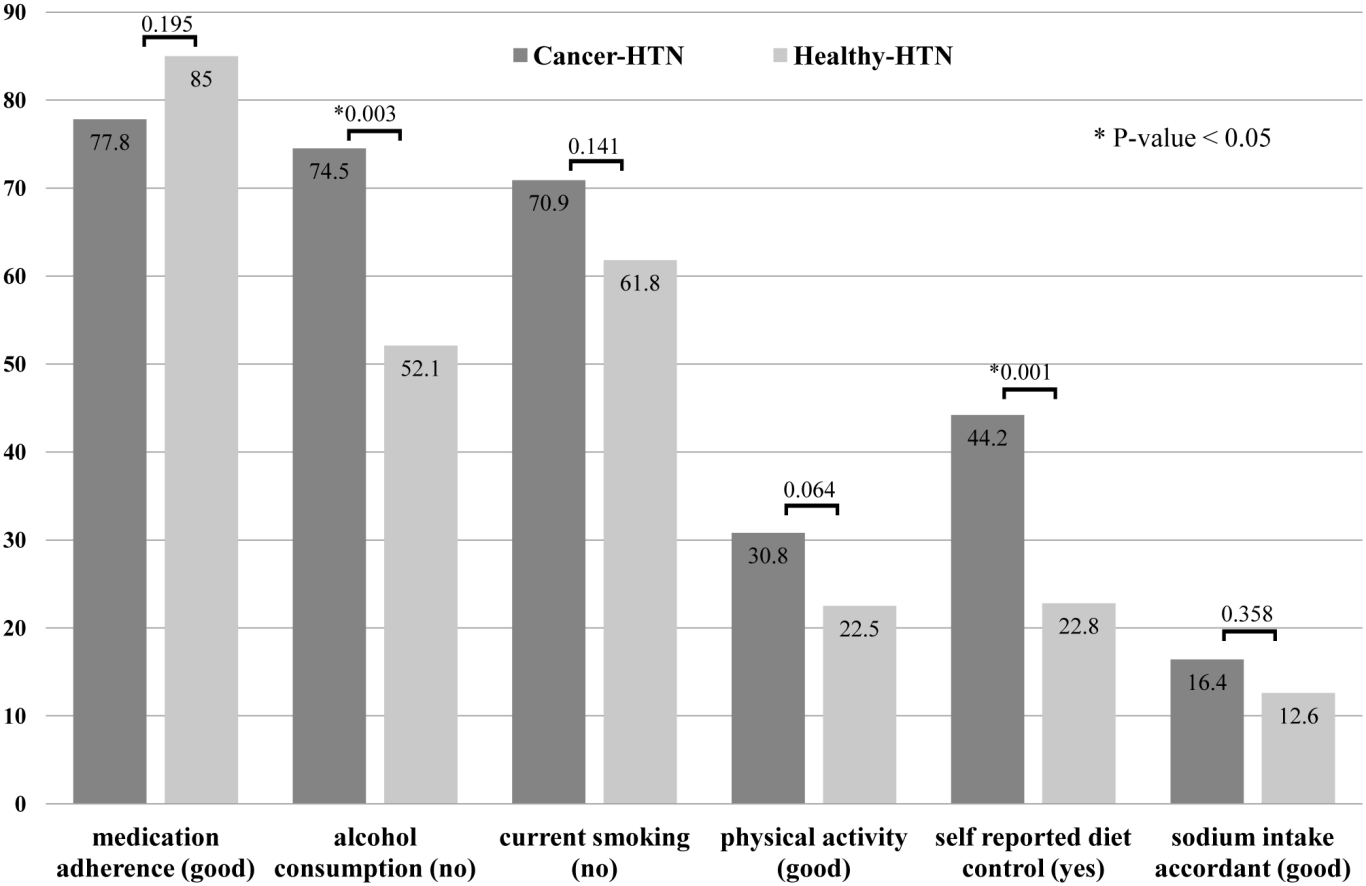
A comparison of health behaviors between the group of hypertensive gastric cancer survivors and the propensity-matched group. The group of hypertensive gastric cancer survivors had lower alcohol consumption. They were more likely to be on dietary control than the control group. However, there was no difference in sodium intake accordance between the two groups.

As shown in Table 3, the cancer survivor group was 2.69 times more likely to be on a diet than the control group (OR: 2.69; 95% CI: 1.44–5.02). According to multivariate analysis, there was a significant difference in dietary control between the two groups (p = 0.001). However, there was no significant difference in sodium intake accordance between the two groups (p = 0.434). The group of hypertensive gastric cancer survivors had significantly (p = 0.003) lower alcohol consumption (OR = 0.30; 95% CI: 0.14–0.66). There was no significant difference in terms of smoking, physical activity, and antihypertensive medication adherence (Table 2 and Fig 2).

**Table 3:** A comparison of health behaviors after propensity matching by a logistic regression analysis.

Variables	Cancer^1^	Noncancer^1^	Unadjusted OR (95% CI)	P-value	Adjusted OR^2^ (95% CI)	P-value
Alcohol consumption						
Yes	24(25.5)	181(47.9)	0.37(0.20–0.69)	0.002	0.30(0.14–0.66)	0.003
No	56(74.5)	219(52.1)	1		1	
Smoking status						
Current	26(29.1)	135(38.2)	0.66(0.37–1.19)	0.168	0.60(0.30–1.19)	0.141
Never/ Former	54(70.9)	265(61.8)	1		1	
Physical activity						
Poor	59(69.2)	308(77.5)	0.65(0.33–1.29)	0.222	0.54(0.28–1.04)	0.064
Good	21(30.8)	91(22.5)	1		1	
Medication adherence						
Poor	16(22.2)	58(15.0)	1.61(0.75–3.47)	0.221	1.66(0.77–3.57)	0.195
Good	64(77.8)	342(85.0)	1		1	
Sodium intake accordance						
Poor	67(83.6)	351(87.4)	0.74(0.34–1.60)	0.436	0.74(0.34–1.60)	0.434
Good	13(16.4)	49(12.6)	1		1	
Self reported dietary control						
Yes	32(44.2)	94(22.8)	2.69(1.44–5.02)	0.002	3.12(1.60–6.10)	0.001
No	48(55.8)	306(77.2)	1		1	

^1^Results were expressed as unweighted numbers (weighted %). Percentages are weighted using the NHANES sampling weights.

OR: Odds ratio; 95%CI: 95% confidence interval to OR.

^2^Adjusted for age, gender, height, and significant covariates (p < 0.1).

## Discussion

Our results revealed that the group of gastric cancer survivors with hypertension showed significantly lower alcohol consumption with more possibility to be on a diet than the control group. However, there was no significant difference in sodium intake accordance or other health behaviors (smoking, physical activity, and medication adherence) between the two groups.

This study examined the health behaviors of gastric cancer survivors with hypertension. Previous studies have generally examined health behaviors without focusing on a specific type of cancer. However, the type of cancer might have considerable influence on health behaviors because they have different mortality and morbidity outcomes depending on the type of cancer. In this regard, this study focused on gastric cancer, the most commonly diagnosed cancer in East Asia with high incidence and survival rates in Korea [2]. To examine whether cancer survivors can sufficiently manage their chronic disease, those participants with history of hypertension were selected because this condition is the most common comorbidity in cancer survivors [2].

In the present study, the group of gastric cancer survivors with hypertension was more likely to be on a diet than the control group. However, there was no significant difference in sodium intake accordance between the two groups. Although there are controversy on the association between higher sodium intake and stomach cancer [19,20], sodium is known as important nutrient in blood pressure control that decreases the risk of stroke and cardiovascular risk [12]. Lowering sodium intake is one of the most important dietary behaviors in hypertensive cancer survivors. According to previous studies of dietary control, 40–47% of cancer survivors improved their dietary habits after being diagnosed with cancer [21,22]. Korean cancer survivors showed higher adherence to diet than those who had never had cancer [23]. But a national cross-sectional survey showed that only 14.8% to 19.1% of cancer survivors engaged in appropriate fruit and vegetable consumption [24]. The remarkable aspect of this study was that 44.2% of cancer survivors answered that they were on diet, but only 16.4% of cancer survivors met the sodium diet accordance. Such result might be explained in the following ways. First, cancer survivors with hypertension may not receive specific instructions from their medical teams on practicing a proper diet to control their high blood pressure. Second, they might have overlooked the importance of diet in controlling high blood pressure but concentrated on diet for cancer. Therefore, primary care physicians should inform hypertensive cancer survivor about appropriate and detailed dietary plan to reduce the risk of cardiovascular disease, therefore improving their overall survival rate, even though they say they have been doing dietary control.

In our study, the cancer survivor group was significantly less likely to consume alcohol than the control group. A total of 25.5% of cancer survivors were current drinkers. Previous studies of cancer survivors' alcohol consumption have produced mixed results. One comparative study in Korea reported that 30.9% of cancer survivors were current drinkers, which was lower than that of the control group [25]. According to a study of U.K. cancer survivors who were diagnosed with cancer under the age of 15, they were less likely to be current drinkers than the general population [26]. On the other hand, a survey based on an Australian population found no significant difference in alcohol consumption between cancer survivors and the general population [27]. One longitudinal study reported that subjects diagnosed with cancer reduced their alcohol intake until the first assessment after their diagnosis. However, this reduction was followed by a slight rebound. Eventually there was no significant difference over the long term [28]. Although previous studies have demonstrated that light-to-moderate drinking can reduce the risk of cardiovascular outcomes [29], cancer survivors' high alcohol intake may also increase the likelihood of a poor prognosis [30,31]. In our study, the cancer survivor group was significantly less likely to consume alcohol than the control group. These discrepancies might be due to the type of cancer. In this regard, future research should consider the type of cancer.

In this study, only 30.8% of gastric cancer survivors with hypertension engaged in sufficient physical activity. There was no significant difference between the two groups. Although this result was consistent with the findings of a previous study based on KHANESIV [25], it was inconsistent with the findings of others that reported some leisure physical activity in the past month for 68.5% of cancer survivors [8] or 30 minutes of brisk walking more than once a week for 69% of breast cancer survivors [32]. The present study's low level of physical activity may be due to the stricter evaluation of cancer survivors based on the most recently updated 2013 ACC/AHA lifestyle management guidelines. Two-thirds of gastric cancer survivors with hypertension did not satisfy the standard level of physical activity in this study. Therefore, physicians should encourage cancer survivors to engage in physical activity.

Previous research has reported that 45%~75% of cancer patients are current smokers at the time of their cancer diagnosis and that 14%-58% continue to smoke even after being diagnosed with cancer [33]. This ratio of current smokers is consistent with the present study's results. Previous studies of smoking among cancer survivors have produced mixed results, as in the case of alcohol consumption. In the present study, there was no significant difference in smoking between the two groups. According to a prospective study of U.K. cancer survivors, the number of current smokers was similar to that for the general population, which is consistent with the results of the present study [28]. However, other studies have suggested that cancer survivors are more [8,9] or less [24,25] likely to smoke than the control group. According to a study in Korea [34], many cancer survivors continued to smoke (17%). Most of them experienced feelings of guilt (75.6%) and censure (77.8%), which led them to conceal their smoking status from healthcare professionals (46.7%) or family members (44.4%) [35]. This suggests that self-report questionnaires may underestimate smoking rates. In any case, 29.1% of gastric cancer survivors with hypertension were current smokers in the present study, indicating a need to inform them that smoking will increase the risk of cancer recurrence as well as the mortality from cardiovascular disease [36].

In this study, medication adherence rate of gastric cancer survivors with hypertension was 77.8%. There was no significant difference between the two groups. The medication adherence rate in the present study was higher than that in previous research (54.4%) [37]. This difference may be explained by the potential overestimation of the medication adherence rate in the present study as a result of data obtained from a survey. In addition, differences in medication adherence standards may lead to differences in adherence rates. Previous research has defined a cumulative medication adherence rate greater than or equal to 80% as appropriate medication adherence [37]. Cardiovascular disease is a common cause of death in cancer survivors, who generally face more cardiovascular risk factors such as obesity, hypertension, and diabetes than the general population [36]. This indicates a need for a better understanding of factors influencing poor medication compliance.

This study contributes to the literature by employing propensity score matching. This method balances case and control groups on a large number of covariates without losing a large number of observations [38,39]. This study is comparable to a quasi-randomized experiment [13]. In addition, the study used data obtained from KNHANES, a nationally representative survey. This study makes a noteworthy contribution by employing data representing cancer survivors at the community level. In addition, this study considered health behaviors of cancer survivors according to gastric cancer. To the best of our knowledge, the present study is the first to focus only on gastric cancer survivors.

This study has some limitations. First, the sample was relatively small. Because the type of cancer may affect the health behavior of cancer survivor, the analysis was focused only on gastric cancer. In this regard, 1:5 propensity score matching was employed to address this limitation. Power calculation could not be carried out because the 1:5 propensity score matching method was used. Future research should consider larger sample. Second, diet-related items in the questionnaire were in a format requiring 24-hour recall and self-reporting. Therefore, diet results may reflect some recall bias. Third, because this research was conducted during 2005–2012 to obtain lager sample, trends in health behaviors might have been changed during this long period. In addition, our results were based only on the Korean population. Therefore, any generalization should be made with caution. In this regard, future research should consider a wider range of countries. Finally, we could not collect the data about cancer status, severity of hypertension, sequence of hypertension, and gastric cancer appearance because no data were collected in the KNHANES. Because these factors play a role in the health behaviors, future research should consider that.

In summary, the group of gastric cancer survivors with hypertension had lower alcohol consumption. They were more likely to be on a diet than the control group. However, there was no significant difference in sodium intake accordance or other health behaviors, including smoking, physical activity or medication adherence between the two groups. Therefore, physicians should advise gastric cancer survivors regarding their comorbidity to better manage their non-cancer diseases and inform them that health behaviors can help reduce the risk of cardiovascular disease and improve their overall survival, even though they say they have been doing health behaviors.
